# Variability in perceived burden and health trajectories among older caregivers: a population-based study in Sweden

**DOI:** 10.1136/jech-2022-219095

**Published:** 2022-12-21

**Authors:** Mariam Kirvalidze, Giorgi Beridze, Anders Wimo, Lucas Morin, Amaia Calderón-Larrañaga

**Affiliations:** 1 Aging Research Center, Department of Neurobiology, Care Sciences and Society, Karolinska Institutet and Stockholm University, Solna, Sweden; 2 Division of Neurogeriatrics, Centre for Alzheimer Research, Department of Neurobiology, Care Sciences and Society, Karolinska Institutet, Solna, Sweden; 3 Inserm CIC 1431, University Hospital of Besançon, Besançon, France; 4 Department of Medical Epidemiology and Biostatistics, Karolinska Institutet, Solna, Sweden; 5 Stockholm Gerontology Research Center, Stockholm, Sweden

**Keywords:** HEALTH, AGING, GERONTOLOGY

## Abstract

**Background:**

The negative effects of informal caregiving are determined by the characteristics of the caregiver-care receiver dyad and the context of care. In this study, we aimed to identify which subgroups of older informal caregivers (1) experience the greatest subjective burden and (2) incur a faster decline in objective health status.

**Methods:**

From a total of 3363 older participants in the Swedish National study on Aging and Care in Kungsholmen (SNAC-K), we identified 629 informal caregivers (19.2%, mean age 69.9 years). Limitations to life and perceived burden were self-reported, and objective health status was quantified using the comprehensive clinical and functional Health Assessment Tool (HAT) score (range: 0–10). Ordered logistic regressions and linear mixed models were used to estimate the associations between caregiving-related exposures and subjective outcomes (cross-sectionally) and objective health trajectories (over 12 years), respectively.

**Results:**

Having a dual role (providing and receiving care simultaneously), caring for a spouse, living in the same household as the care receiver and spending more hours on caregiving were associated with more limitations and burden. In addition, having a dual role (β=−0.12, 95% CI −0.23 to −0.02) and caring for a spouse (β=−0.08, 95% CI −0.14 to −0.02) were associated with a faster HAT score decline. Being female and having a poor social network were associated with an exacerbation of the health decline.

**Conclusions:**

Both the heterogeneity among caregivers and the related contextual factors should be accounted for by policymakers as well as in future research investigating the health impact of informal caregiving.

**WHAT IS ALREADY KNOWN ON THIS TOPIC**

- Informal caregiving has been shown to have negative effects on those providing care. However, when it comes to older caregivers, the available evidence is not consistent.

**WHAT THIS STUDY ADDS**

- By using longitudinal data with up to 12 years of follow-up, we estimated the association between informal caregiving and both subjective (burden, limitations) and objective (health status) outcomes in older adults.

**HOW THIS STUDY MIGHT AFFECT RESEARCH, PRACTICE OR POLICY**

- Our findings emphasise the need to target public health interventions to subgroups of older caregivers who may be at an increased risk of experiencing the negative impact of caregiving.

## Introduction

In the context of population ageing and the resulting overall increase in care needs, informal care is becoming a large contributor to societal welfare worldwide. An informal caregiver is defined as any relative, partner, friend or neighbour who has a significant personal relationship with and provides a broad range of assistance for, an older person with a chronic or disabling condition.^1^ The relationship between provision of informal care and the overall well-being of caregivers is inherently complex. Providing informal care is often viewed by relatives of older adults as a meaningful and fulfilling experience, and, in some contexts, may even prolong longevity.^2^ On the other hand, informal caregiving has been shown to increase the carers’ feelings of stress and burden, leading to negative health-related outcomes.^3 4^ Given the current European-wide shift from institutional to community-based care,^5^ the number of adult children, spouses and partners providing informal care to older relatives is expected to significantly increase in the years ahead.^6^

Sweden is no exception. The number of informal caregivers has grown fast over the past two decades, contributing to almost two-thirds of the care provided to community-dwelling older people.^7^ Moreover, about a quarter of intrahousehold caregivers are now 75—84 years old, and the number of caregivers is increasing at an especially fast pace among the oldest old.^8^ These caregivers might be more vulnerable to the potential negative impacts of caregiving, partly due to their own age-related problems and the physical and mental health risks generally associated with providing informal care.^3 4 9^ However, both the characteristics of the caregiver-care receiver dyad and the context of care seem to play an important role in determining who is most affected by the collateral effects of informal caregiving. For instance, the nature of the relationship between the caregiver and the care receiver (eg, spouse, child, neighbour), the intensity of caregiving, the availability of support networks and services and the health literacy of informal caregivers have been found to either buffer or magnify the perceived burden of caregiving.^10–12^

Despite the considerable body of evidence describing the different factors that may modulate the association between providing informal care and poorer-than-average health outcomes, findings are largely contradictory when it comes to older caregivers. Some studies report that older people might be highly vulnerable to the negative consequences of informal caregiving,^13^ while others suggest that they are well equipped for the role.^14 15^ These contradictory findings might be explained by the methodological limitations of observational studies, such as the low participation rate of older adults in surveys, convenience sampling, short follow-up time and the potential for reverse causality (ie, selection of healthier older adults into informal caregiving). The discrepancies in published estimates may also be explained by the high heterogeneity of older informal caregivers, not only in terms of baseline health status but also in terms of coping strategies and capacity for resilience.^16 17^

The lack of high-quality, longitudinal data about the potentially detrimental effect of informal caregiving in old age is hindering our understanding of the problem and preventing us from tailoring support interventions that address the needs of the most affected caregivers. In this descriptive study based on a population-based Swedish cohort with up to 12 years of follow-up, our goals were to identify which subgroups of older informal caregivers: (1) experience most limitations to life and perceive more burden and (2) incur a faster decline in objective health status.

## Methods

### Study population

We used data from the Swedish National study on Aging and Care in Kungsholmen (SNAC-K; http://www.snac-k.se), a longitudinal study of randomly selected adults aged 60 years or older living at home or in institutions in the Kungsholmen district of Stockholm, Sweden. Baseline population was recruited in 2001–2004 and included a total of 3363 individuals (73.3% participation rate) that were followed up regularly; every 6 years for the younger cohorts (<78 years) and every 3 years for the older cohorts (≥78 years). Participants underwent comprehensive clinical examinations, interviews and assessments by physicians, nurses and psychologists at each study visit. Participants who answered positively to the question ‘Do you provide care to a friend or a relative?’ during nurse-led interviews were defined as self-identified informal caregivers. These participants went on to complete the rest of the caregiver-related questions, some of which we use as exposures or outcomes in this study.

### Outcomes

#### Limitations to life and perceived burden

Limitations to life and perceived burden were assessed using two questions from the face-to-face interview with a trained nurse. Participants were first asked ‘How has your ability to live your own life, preserve your personal relationships, and enjoy your leisure time been affected by your situation as a caregiver?’ and responded by using a 4-point Likert scale ranging from ‘no limitations’ to ‘great limitations’. Participants were then asked ‘Overall, how often do you feel burdened by caring for your dependent?’ and responded using a 5-point Likert scale ranging from ‘never’ to ‘almost always’.

#### Objective health status

Caregivers’ objective health status was assessed using the Health Assessment Tool (HAT), a clinical and functional assessment tool that has been specifically designed for—and validated in—cohorts of older people.^18^ HAT incorporates five indicators: physical function (measured through participants’ walking speed), cognitive status (assessed through the Mini Mental State Examination), multimorbidity (defined as the number of coexisting chronic diseases from a list of 60 chronic conditions) and limitations in basic and instrumental activities of daily living (ADL, IADL). The resulting score ranges from 0 (poor health) to 10 (good health). Detailed information on this measure is available elsewhere.^18^

### Exposures

The exposures in this study were selected based on a thorough review of the existing literature on caregiver burden^10–12 19^ and the availability of data in SNAC-K. We, thus, characterised caregivers based on whether they had a *dual role* (defined as simultaneously receiving informal or formal care and providing informal care), whether they provided care to a spouse, whether they lived in the same household as the care receiver, and the number of hours of care that they provided per month. The variable related to the number of hours of care was derived from a series of questions asked by the nurse (how many hours per typical day, days per week and weeks per month did the participant provide care for). The continuous variable was split into three categories: 0 hours (10% of caregivers), 1–30 hours (60% of caregivers) or ≥30 hours (30% of caregivers) per month. The decision on cut-offs was based on the distribution of the reported data and discussions between the authors in order to identify meaningful groups of caregivers, reflecting the intensity of their caregiving roles. Of note, a small number of participants responded positively to the question ‘Do you provide care to a friend or a relative?’ but reported not having provided any care (in hours) during a month prior to the interview. These participants were included in the analyses since they self-identified as caregivers despite providing only occasional care.

### Covariates and potential effect modifiers

Covariates included in our study were age, sex and highest educational attainment (primary school, high school, university). The latter sociodemographic variables as well as social network (poor, moderate, rich) were tested for their potential effect-modifying effects. Social network was operationalised using several items related to social connections and social support, such as the frequency of direct or remote contacts with parents, children, relatives, neighbours and friends as well as the satisfaction with these contacts.^20^

### Statistical analysis

**Figure 1:**
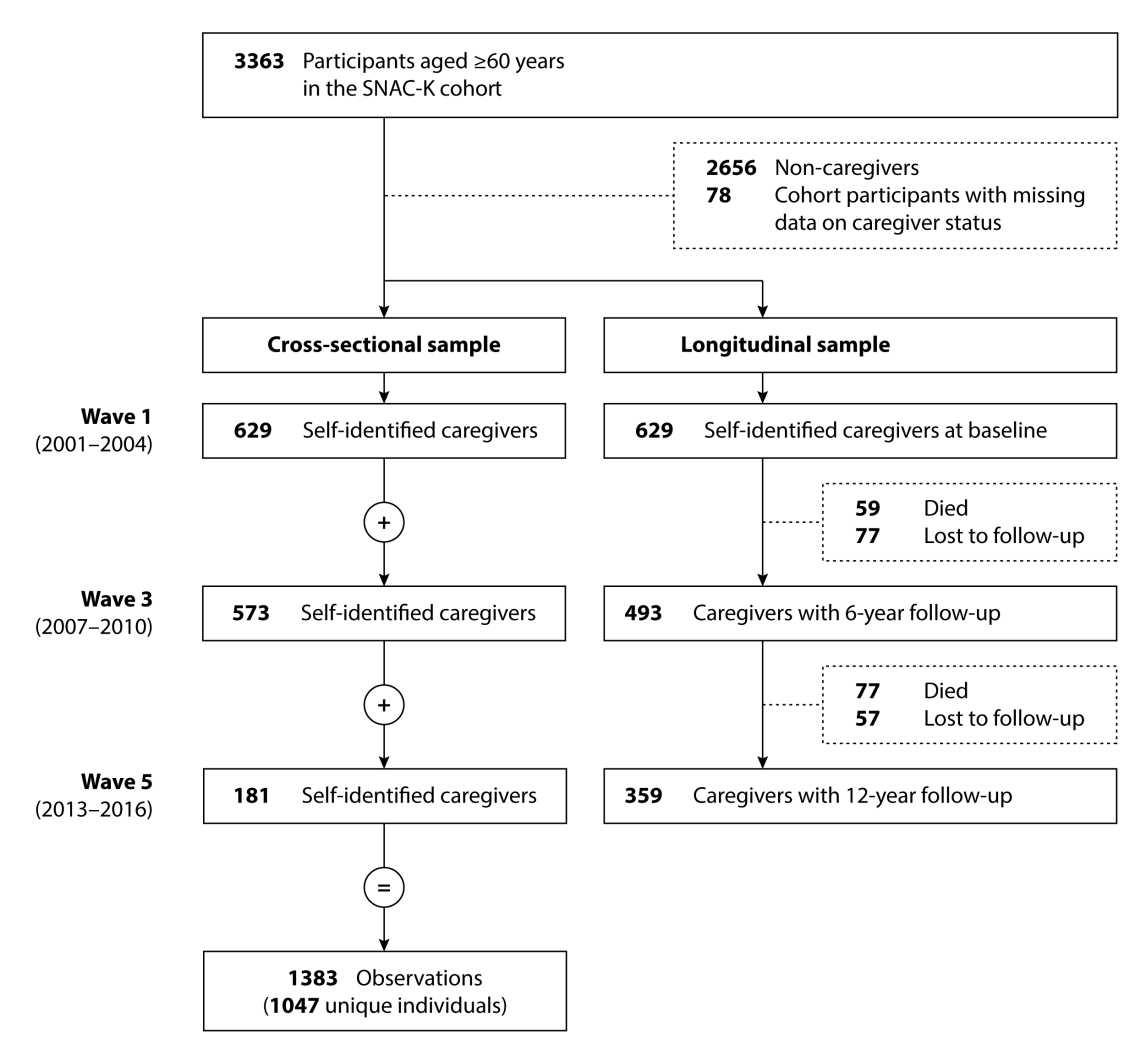
Flowchart of study population for cross-sectional and longitudinal samples. SNACK-K, Swedish National study on Aging and Care in Kungsholmen.

Limitations to life and perceived burden were analysed among informal caregivers who attended the SNAC-K study visit/examination at three different timepoints: at wave 1 (2001–2004, n=629), at wave 3 (2007–2010, n=573) and at wave 5 (2013–2016, n=181). This part of our analysis relates to the cross-sectional sample described in the study flowchart (figure 1). Ordered logistic regression models with clustered SEs were used to estimate the cross-sectional associations between the exposures of interest and the two self-reported outcomes (limitations to life, burden), while adjusting for age, sex and level of education. Interactions between exposure variables and age, sex, education and social network were examined. Results are reported as proportional ORs with 95% CIs. Indicator variables were constructed to cross-classify individuals across caregiving-related and sociodemographic characteristics.

We then investigated trajectories in objective health status among the 629 informal caregivers who entered the SNAC-K cohort at baseline (namely in 2001–2004) and who were followed prospectively for an average period of 12 years (see the longitudinal sample described in the study flowchart, figure 1). Adjusted linear mixed models with random intercepts were used to obtain β coefficients and 95% CIs for the association between each caregiving-related exposure and the observed changes in the HAT score over the follow-up period. The interaction term between the exposures and years since cohort entry was included as a fixed effect, so that a negative β coefficient indicates a steeper decline in health status over time. We examined three-way interactions between each exposure that was significant in the main model, follow-up time and sociodemographic variables (age, sex, education and social network). Inverse probability weighting (IPW) was used to account for attrition due to death and dropout. Statistical analyses were performed in StataSE V.15 (StataCorp LLC, College Station, Texas). The level of statistical significance was set at p<0.05 for the main analyses and at p<0.1 for the interaction analyses.

## Results

At baseline, 629 of 3363 (19.2%) SNAC-K participants identified themselves as caregivers; the mean age of caregivers was 69.9 years, 66% were women and 40.5% had university education. Dual role and spousal caregiving were reported by 6.4% and 11.9% of the baseline caregiver sample, respectively. Of note, 28.4% of caregivers reported suffering from at least some limitation to life and 27.6% reported experiencing at least some burden related to caregiving. Detailed demographic characteristics as well as the distribution of exposures and outcome measures in each SNAC-K wave are reported in table 1.

**Table 1:** Characteristics of the study population and outcomes distribution

	SNAC-K wave 1* 2001–2004	SNAC-K wave 3 2007–2010	SNAC-K wave 5 2013–2016
	n=629 out of 3285 (19.2%)	n=573 out of 2012 (28.5%)	n=181 out of 1272 (14.2%)
Age, mean (SD)	69.9 (8.5)	72.2 (6.7)	77.4 (5.8)
Sex, women (%)	415 (66.0%)	375 (65.4%)	112 (61.9%)
Education (%)			
Elementary	71 (11.3%)	42 (7.3%)	12 (6.6%)
High school	303 (48.2%)	240 (41.9%)	91 (50.3%)
University	255 (40.5%)	291 (50.8%)	78 (43.1%)
Dual role (%)	40 (6.4%)	50 (8.7%)	12 (6.6%)
Caring for spouse (%)	75 (11.9%)	82 (14.3%)	84 (46.4%)
Living in same household (%)	85 (13.5%)	80 (14.0%)	75 (41.4%)
Hours of care per month (%)			
0	38 (7.0%)	82 (16.0%)	9 (5.9%)
1–30	353 (64.7%)	276 (53.9%)	106 (69.7%)
30+	155 (28.4%)	154 (30.1%)	37 (24.3%)
Limitations to life† (%)			
None	414 (71.6%)	418 (74.4%)	107 (60.1%)
Slight	87 (15.1%)	78 (13.9%)	35 (19.7%)
Moderate	52 (9.0%)	39 (6.9%)	21 (11.8%)
Great	25 (4.3%)	27 (4.8%)	15 (8.4%)
Perceived burden‡ (%)			
Never	417 (72.4%)	440 (79.3%)	98 (57.3%)
Rarely	41 (7.1%)	47 (8.5%)	27 (15.8%)
Sometimes	70 (12.2%)	28 (5.0%)	28 (16.4%)
Quite often	25 (4.3%)	25 (4.5%)	15 (8.8%)
Almost always	23 (4.0%)	15 (2.7%)	3 (1.8%)
HAT score, mean (SD)	8.3 (1.2)	7.5 (1.4)	7.5 (1.2)

Missing at baseline (wave 1): hours of care per month=84, limitations to life=51, perceived burden=53, HAT=19. Missing in wave 3: hours of care per month=61, limitations to life=11, perceived burden=18, HAT=13. Missing in wave 5: hours of care per month=29, limitations to life=3, perceived burden=10, HAT=3.

*Wave 1 constitutes the longitudinal sample within our study (see figure 1).

†Refers to the question from the nurse interview on the magnitude of perceived limitations to life, caused by caregiver activities.

‡Refers to the question from the nurse interview on the frequency of perceived burden of caregiving.

**Figure 2:**
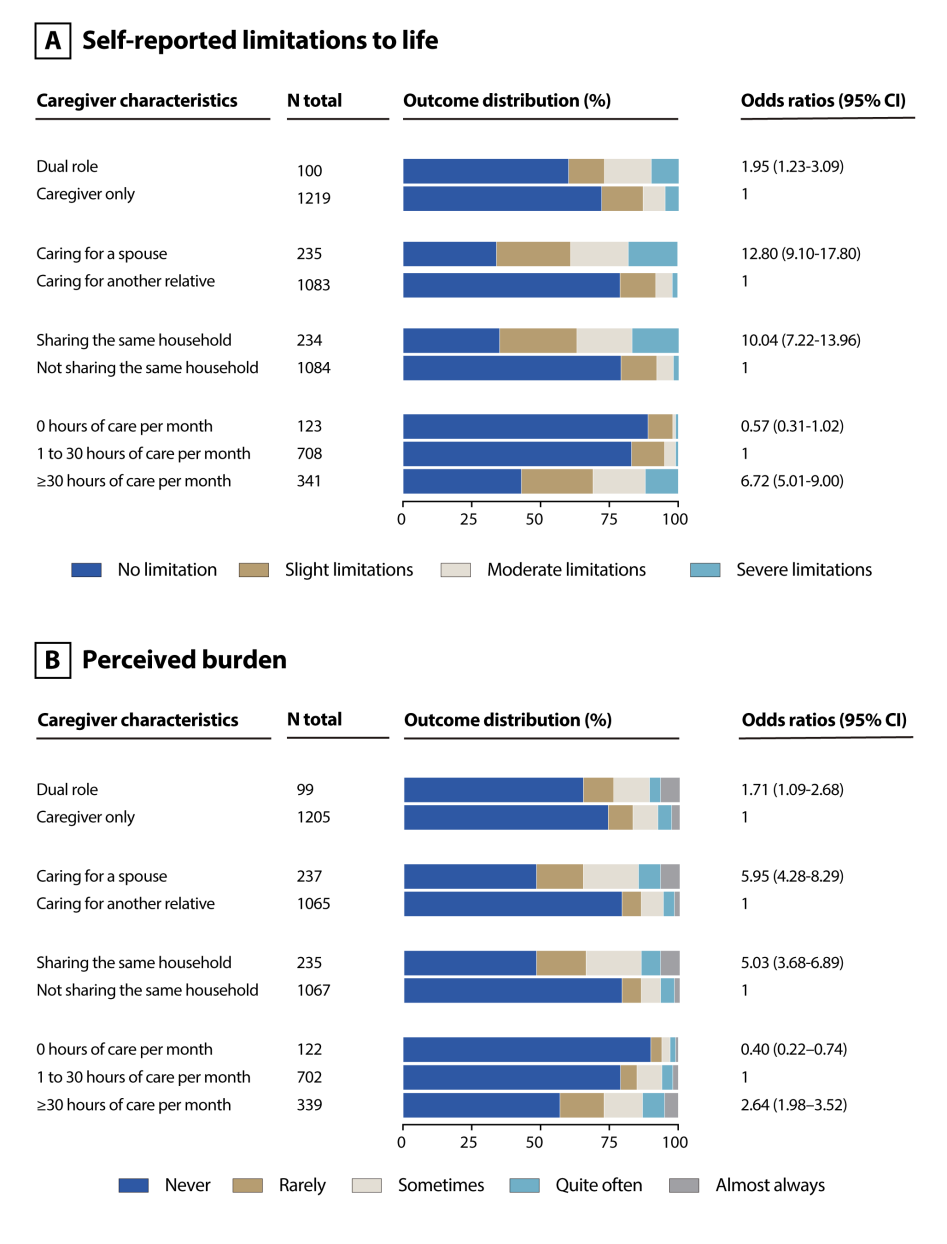
Cross-sectional sample: association of caregiving factors with self-reported limitations to life (A) and perceived burden (B). Models adjusted for age, sex, education.

HAT, Health Assessment Tool;SNACK-K, Swedish National study on Aging and Care in Kungsholmen;

As shown in figure 2, having a dual role, caring for a spouse, living in the same household and providing more than 30 hours of care per month were associated with a higher number of self-reported limitations to caregivers’ lives. For the outcome related to perceived burden, having a dual role, caring for a spouse, living in the same household and providing more than 30 hours of care per month were associated with higher levels of burden, while not having provided any care in the previous month was associated with lower burden.

We further explored interactions between exposures and sociodemographic characteristics of caregivers against subjective measures of health (see online supplemental figure 1). For almost all caregiver-related exposures (ie, spousal care, intrahousehold care and provision of more than 30 hours of care), higher age, female sex and higher level of education were significantly associated with more limitations to life and a higher perceived burden.

10.1136/jech-2022-219095.supp1 Supplementary data

**Figure 3:**
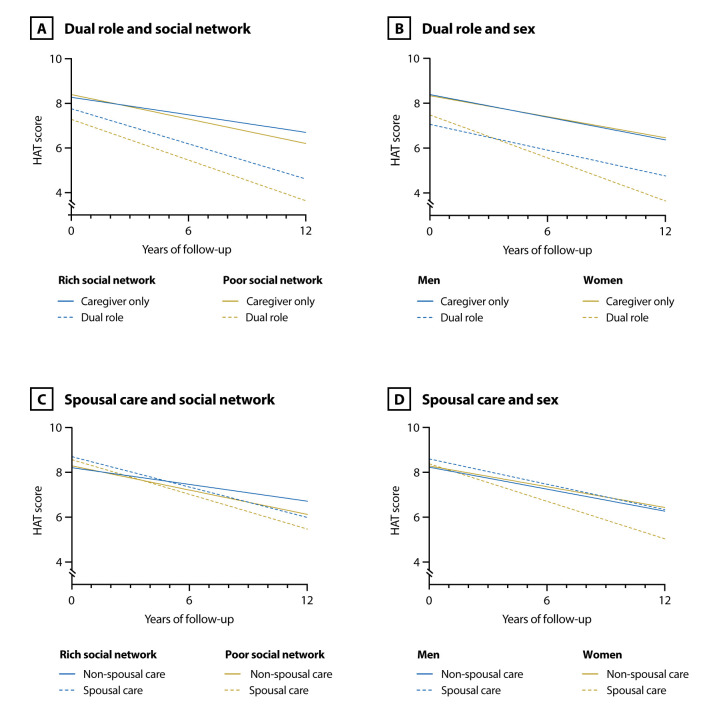
Longitudinal sample: estimated changes in objective health status (ie, HAT score) throughout the 12-year follow-up according to indicator variables cross-classifying caregiving factors (ie, dual role [A, B] and spousal care [C, D]) and sociodemographic characteristics (ie, social network [A, C] and sex [B, D]). Models adjusted for age, sex, education. Inverse probability weighted models. HAT, Health Assessment Tool

As shown in table 2, having a dual role and caring for a spouse at baseline were associated with a faster average annual decline of the HAT score over the 12- year period. This means that, for example, caregivers with a dual role had an average annual score decline of 0.12 points higher than those with no dual role (adjusting for caregivers’ age, sex and education). We did not observe statistically significant three-way interactions between caregiving variables, follow-up time and sociodemographic variable. Figure 3 (online supplemental table 1) shows the trajectories of HAT scores according to indicator variables. Among dual caregivers, women and those with a poorer social network incurred faster declines in HAT scores. Similarly, among spousal caregivers, women and participants with poorer social network showed steeper declines in HAT scores.

**Table 2:** Longitudinal sample: association between baseline caregiving factors and average annual rate of health decline (ie, HAT score) during the 12-year follow-up

	β coefficient (95% CI)
Dual role*	−0.12 (−0.23 to −0.02)
Caring for spouse*	−0.08 (−0.14 to −0.02)
Living in same household*	−0.05 (−0.11 to 0.01)
Hours of care per month	
0 vs 1–30	−0.01 (−0.08 to 0.08)
30+ vs 1–30	−0.02 (−0.06 to 0.02)

*Yes versus no.

HAT, Health Assessment Tool.

## Discussion

In this population-based study of older adult caregivers in central Stockholm, we found that having a dual role, caring for a spouse, cohabiting with the care receiver and a more intensive caregiving activity were significantly associated with more self-reported limitations and burden to caregivers’ lives. In addition, having a dual role and caring for a spouse were associated with a faster decline in objective health. Some of these associations were moreover moderated by different sociodemographic factors, namely, age, sex, education and social network.

Our findings are largely in line with previous research on older informal caregivers. It has, for instance, been suggested that certain subgroups of caregivers such as female spouses or persons residing in the same households as care receivers incur more negative effects of informal caregiving.^10^ In a recent Swedish national survey, spousal carers provided more care, more frequently, and experienced a higher negative impact on their social life and psychological and physical health.^21^ In a sample of older caregivers derived from the UK census, elderly frail spousal caregivers and middle-aged women with multiple roles were most susceptible to the negative health effects of caregiving.^22^ However, studies on intrahousehold caregivers are very liable to selection bias. According to a pan-European longitudinal study, intrahousehold caregivers reported worse health, while caregivers from outside the household reported even better health than non-caregivers.^23^ This correlation was deemed to be largely due to selection into caregiving, whereby sicker people are more likely to provide care within the household while people in better health are more likely to do so outside the household.^23^

Compared with the above-mentioned factors, the dual role of caregivers had not been explored to the same extent. Yet, this situation is becoming more common. Declining family size, increased geographical mobility, postponement of the retirement age and rising female participation in labour markets have led to an increase in the average age of informal caregivers,^24^ who are likely to require social formal or informal care themselves. Data from the UK indicate that numbers of older caregivers are increasing more rapidly than younger age groups,^25^ a phenomenon that may accelerate the rate of new dual-role caregivers in the near future. In our sample of caregivers, 6.4% had a dual role at baseline, which is consistent with findings from a cross-sectional sample of older adults in Ireland, where 5% of older caregivers reported also receiving care.^26^ This subgroup of informal caregivers seems to be particularly vulnerable to negative health outcomes because they themselves require care.

There are several explanations as to why and how informal caregiving may impact subjective feelings of limitations to life and burden. Spousal and intrahousehold caregivers might perceive a higher burden due to the so-called *family effect*, that is, the impact of being worried about someone close irrespective of providing care or other factors (intensity, type). Increasing hours of care provision understandably leads to more limitations to life, since caregiving time competes with time that would otherwise be spent on leisure or work activities. Accordingly, in a sample of Dutch informal caregivers, well-being was significantly associated with the health of the care receiver (*family effect*) and the number of caregiving tasks (*caregiving effect*).^27^ As for dual-role caregivers, it is expectable that assuming caregiver and care receiver roles simultaneously will lead to exponentially higher feelings of limitations and burden. These older adults are already limited and burdened by their own needs and might, thus, perceive caregiving as more challenging. Our unexpected finding that caregivers with lower education report less subjective burden may be due to the fact that this group of carers experience less disruptions to their career and leisure because those activities were already limited even prior to assuming the role of an informal caregiver.

There are equally plausible theories that might elucidate the associations between caregiving factors and objective health, as observed in our study. According to the caregiver stress model,^28^ informal care provision is associated with significant stress that leads to psychological distress and, eventually, to depression and anxiety. Such physiological changes involve the sympathetic nervous system and cortisol overproduction and may promote the development of various disease processes. Being a caregiver might moreover interfere with care-seeking behaviours, such as getting timely medical advice and maintaining optimal levels of physical activity and good nutrition. Spousal caregivers are more likely to provide care more frequently^21^ and are, thus, susceptible to adopting worse health behaviours, in addition to the strain that it exerts on the caregiving process.

Social network seems to have an important role in determining the speed of health decline among older caregivers with spousal and/or dual roles. Social isolation and lack of social connectedness among older adults have been associated with worse health outcomes,^29^ possibly due to unfavourable health behaviours, that is, older adults with rich social network are more motivated and more often urged to take care of their health and maintain healthy lifestyle. Having a rich and supportive social network might also help distribute the obligations of caregiving and alleviate some of the emotional burden caused by the caregiving responsibility.

Steeper health declines were also seen among women with spousal and dual roles, which might be explained by women providing more extensive care.^30^ Compared with their male counterparts, female caregivers report providing more hours of care,^22^ higher caregiver burden^31^ and more disease symptoms.^13^ Of note, a systematic review on gender differences in caregiver health reported differences in depression and physical health of caregivers to be larger than those found in the general population.^30^ Another possible explanation underlying gender differences may be related to the willingness to accept support. It has been suggested that, since male caregivers are less comfortable with the caregiving role, they are more inclined to seek and receive outside assistance for caregiving from formal and informal sources.^32 33^ In other words, their caregiving experience might be ‘lighter’ because of the lower intensity of tasks and more support being sought. However, this remains contested since evidence on gender differences in the use of support services is conflicting, and gender roles have significantly shifted since the start of this debate.

### Strengths and limitations

There are several strengths to this study. The SNAC-K cohort entails longitudinal data covering a wide range of sociodemographic and physical and mental health-related measures with up to 12 years of follow-up. This enabled us to account for different sources of heterogeneity among older caregivers.^34^ Unlike many previous caregiving studies that relied on non-probabilistic samples, SNAC-K participants were randomly selected from the reference population, which increases the generalisability of our findings.^35^ Our study has shown that both subjective (perceived limitations to life and burden) and objective outcomes (health status) are influenced by similar caregiving factors, which strengthens our findings and supports the reliability of subjective measures for predicting objective effects across various caregiver subgroups.

Nevertheless, our study needs to be interpreted considering several limitations. We lack information on several care receiver-related aspects that might play a role in these associations, such as their illness (ie, type, stage and progression trajectory), their relationship with the caregiver (eg, reciprocity and intimacy, shared values, etc) and other sources of formal care that could potentially alleviate caregivers’ burden. Thus, given the important residual confounding related to the caregiving experience, and the inherent limitation of reverse causation in cross-sectional and even non time-lagged longitudinal analyses, findings from our analyses are limited to the descriptive, associational framework. Surveys or questionnaires built specifically for capturing the caregiving experience would be better fitted to determine potential causal pathways. The generalisability of our findings beyond the reference population is limited. The Kungsholmen district of Stockholm is an affluent urban area, with predominantly white population of Swedish descent. Therefore, socioeconomic as well as racial/ethnic diversity of caregivers could not be taken into consideration in this study.

### Conclusions

Informal caregiving is a complex experience, shaped by factors related not only to the caregivers but also to the care receivers and the broader context in which the caregiving relationship takes place. Our findings suggest that older informal caregivers who also need care (dual role) and those who care for their spouse are more susceptible to reporting subjective burden and experiencing worse health trajectories over time. Women and caregivers with a poor social network are especially vulnerable to these negative outcomes. Further research is warranted to fully capture the heterogeneity among caregivers also taking contextual factors into account. Nonetheless, our findings emphasise the importance of differentiating public health efforts by subgroups of caregivers. Policymakers should indeed avoid taking a ‘one-size-fits-all approach’ when designing support interventions to older caregivers and should instead aim to target subgroups of caregivers who experience the largest negative impact of informal caregiving.

## Data Availability

Data are available upon reasonable request. Access to SNAC-K data is available to researchers with approval from the SNAC-K data management and maintenance committee. Applications for accessing SNAC-K data can be submitted to Maria Wahlberg (maria.wahlberg@ki.se) at the Aging Research Center, Karolinska Institutet.
