# Supplementary material for: Variability in perceived burden and health trajectories among older caregivers: a population-based study in Sweden
Source: J Epidemiol Community Health. 2022 Dec 21;77(2):125–32. doi: 10.1136/jech-2022-219095 (PMC9872244; doi:10.1136/jech-2022-219095)
Supplement: Supplementary data [file jech-2022-219095supp001.pdf]

Supplementary Figure 1. Association of indicator variables cross-classifying caregiving factors (i.e., spousal care, cohabitation between caregiver and care receiver, intensity of caregiving) and sociodemographic characteristics (i.e., age, sex, education) with self-reported limitations to life and perceived burden. Models* adjusted for age, sex, education.

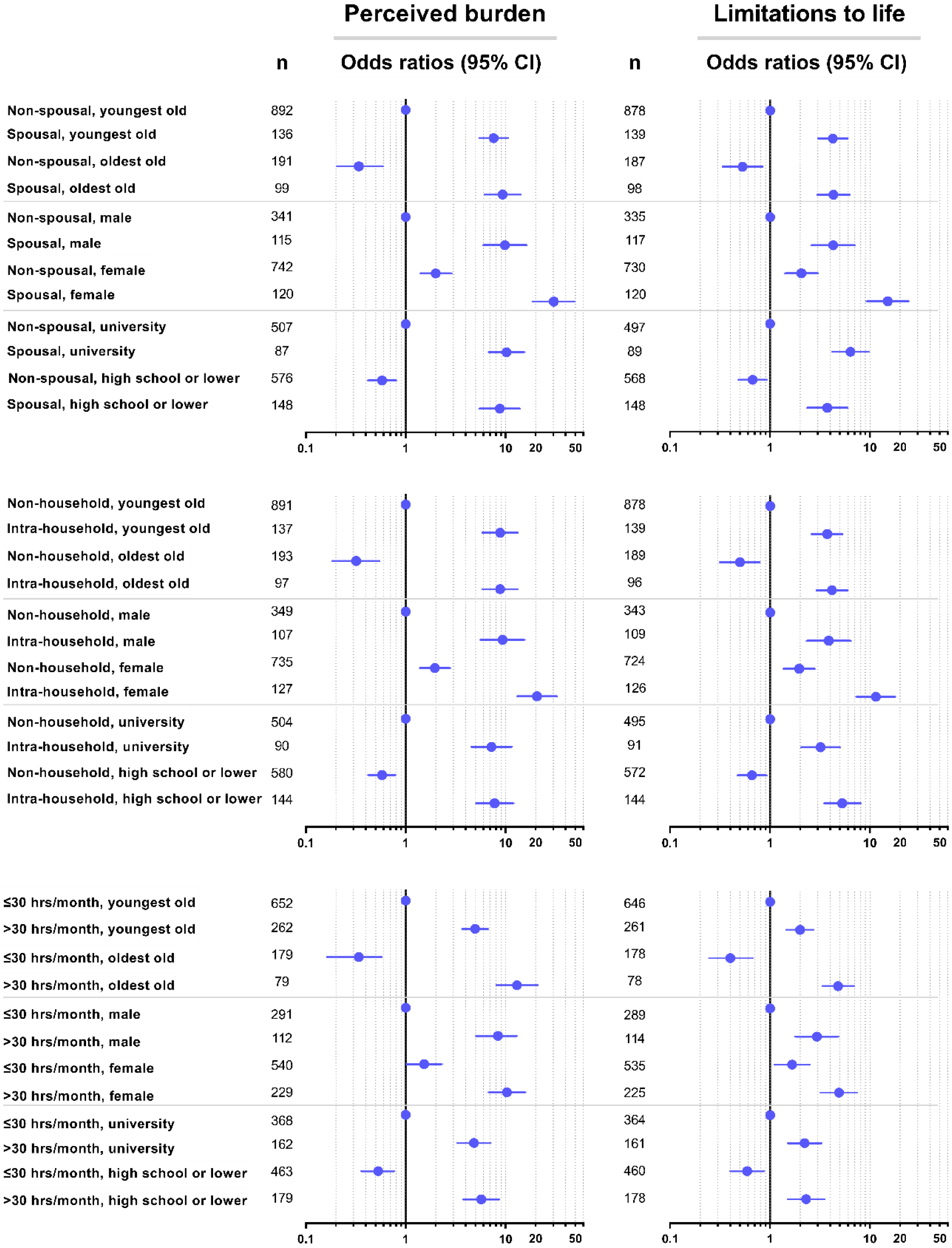

*Independent models.

Supplementary Table 1. Association between baseline indicator variables cross-classifying caregiving factors (i.e., dual role and spousal care) and sociodemographic characteristics (i.e., social network and sex) and annual rate of health decline (i.e., HAT score) during the 12-year follow-up. Models adjusted for age, sex, education. Inverse probability weighted models.

	**β coefficient (95% CI)**
**Dual role and social network**	
Caregiver only, rich social network	Reference
Dual role, rich social network	-0.13 (-0.43; 0.16)
Caregiver only, poor social network	-0.05 (-0.09; -0.02)
Dual role, poor social network	-0.17 (-0.28; -0.06)
**Dual role and sex**	
Caregiver only, male	Reference
Dual role, male	-0.02 (-0.25; 0.21)
Caregiver only, female	0.01 (-0.02; 0.05)
Dual role, female	-0.15 (-0.26; -0.04)
**Spousal care and social network**	
Non-spousal care, rich social network	Reference
Spousal care, rich social network	-0.10 (-0.19; -0.02)
Non-spousal care, poor social network	-0.06 (-0.09; -0.02)
Spousal care, poor social network	-0.13 (-0.21; -0.05)
**Spousal care and sex**	
Non-spousal care, male	Reference
Spousal care, male	-0.03 (-0.10; 0.05)
Non-spousal care, female	0.01 (-0.03; 0.05)
Spousal care, female	-0.12 (-0.21; -0,02)
